# Systematic review and meta-analysis comparing outcomes of multi-port versus single-incision laparoscopic surgery (SILS) in Hartmann’s reversal

**DOI:** 10.1007/s00384-024-04752-2

**Published:** 2024-11-28

**Authors:** Omar E. S. Mostafa, Shafquat Zaman, William Beedham, Georgios Kakaniaris, Najam Husain, Lalit Kumar, Akinfemi Akingboye, Peter Waterland

**Affiliations:** 1 Department of General and Colorectal Surgery, Russells Hall Hospital, Dudley Group NHS Foundation Trust, Dudley, UK; 2 Department of General and Colorectal Surgery, Queen’s Hospital Burton, University Hospital of Derby and Burton NHS Foundation Trust, Derby, UK; 3 College of Medical and Dental Sciences, University of Birmingham, Edgbaston, Birmingham, UK; 4 College of Health and Life Sciences, Aston University, Birmingham, UK

**Keywords:** Laparoscopy, SILS, Multi-port, Colostomy reversal

## Abstract

**Background:**

Colostomy formation as part of the Hartmann’s procedure is often performed during emergency surgery as a damage limitation measure where attempts at bowel anastomosis and continuity are contraindicated. Hartmann’s reversal (HR) remains challenging and can be attempted through open surgery and various minimally invasive techniques (laparoscopic and robotic platforms). We aimed to analyse outcomes of conventional multi-port laparoscopy (CL) versus single-incision approach (SILS) in patients undergoing HR.

**Methods:**

A comprehensive online search of various databases was conducted in accordance with PRISMA guidelines including Medline, PubMed, Embase, and Cochrane. Comparative studies of patients undergoing CL and SILS for HR were included. Analysed primary outcomes were total operative time and mortality rate. Secondary outcomes included post-operative complications, length of hospital stay, risk of visceral injury intra-operatively, and re-operation rate. Combined overall effect sizes were calculated using the random-effects model, and the Newcastle–Ottawa Scale (NOS) was used to assess bias.

**Results:**

Two observational studies matching our inclusion criteria with a total of 160 patients (SILS 100 vs. CL 60) were included. Statistical difference was observed for one outcome measure: operative duration (MD − 44.79 CI − 65.54– − 24.04, *P* < 0.0001). No significant difference was seen in mortality rate (OR 1.66 CI 0.17–16.39, *P* = 0.66), overall post-operative complications (OR 0.60 CI 0.28–1.32, *P* = 0.20), length of stay (MD − 0.22 CI − 4.25–3.82, *P* = 0.92), Clavien-Dindo III + complications (OR 0.61 CI 0.15–2.53, *P* = 0.50), risk of visceral injury (OR 1.59 CI 0.30–8.31, *P* = 0.58), and re-operation rates (OR 0.73 CI 0.08–6.76, *P* = 0.78).

**Conclusion:**

Accounting for study limitations, the SILS procedure seems to be quicker with non-inferior outcomes compared with the conventional multi-port approach. This may lead to better patient satisfaction and cosmesis and potentially reduce the risk of future incisional hernia occurrence. However, well-designed, randomised studies are needed to draw more robust conclusions and recommendations.

## Introduction

First described in 1921, the Hartmann’s procedure (HP) or proctosigmoidectomy is used to safely perform rectosigmoid resections in clinically unstable, morbid, or grossly septic patients as a therapeutic intervention and damage control measure. Often used during emergency surgery, HP can be used to treat various acute colorectal pathology [1] in instances where immediate anastomosis or restoration of bowel continuity is considered high-risk. Rarely HP can be used palliatively in patients with advanced colorectal malignancy to relieve impending colonic obstruction.

A hallmark of the procedure following rectosigmoid resection and closure of the anorectal stump is the formation of a stoma (end colostomy). This obviates the need for colorectal anastomosis and improves intra- and post-operative recovery [1, 2]. However, the presence of a colostomy (or any stoma) can be associated with significant psychological and physical burden/trauma for patients. Consequently, following a full recovery, suitable patients may be offered or considered for colostomy reversal surgery/Hartmann’s reversal (HR) [3, 4]. HR remains technically challenging particularly in cases involving extensive adhesions following peritonitis and as such is associated with significant morbidity and mortality [1, 5].

Colostomy reversal surgery can be performed through either an open approach or various minimally invasive techniques including conventional laparoscopy and robotic platforms. The exact surgical strategy will depend on a number of factors including surgeons’ expertise and experience and patient-related factors.

The traditional open surgery approach for colostomy reversal is associated with high rates of surgical site infection (SSI), increased length of hospital stay (LOS), and post-operative complications compared with laparoscopic reversal [6, 7]. Advancements in laparoscopic techniques and technologies have led to the development of single-incision laparoscopic surgery (SILS) [8], which has proven efficacious in malignant colorectal and upper gastrointestinal surgery, with shorter hospital stay and lower risk of incisional hernias [9, 10]. Non-comparative studies of SILS in HR have also demonstrated safety of this technique together with a low-risk profile [11, 12].

While the literature continues to support the laparoscopic approach over open HR, there remains little evidence on whether the single-port approach (SILS) is clinically superior to the conventional, multi-port laparoscopic technique (CL). We therefore conducted a systematic review and meta-analysis of the available evidence to evaluate clinical outcomes and compare these techniques.

## Materials and methods

This systematic review and meta-analysis was conducted in accordance with the Cochrane Handbook for Systematic Reviews and Meta-Analyses [13], the Preferred Reporting Items for Systematic Reviews and Meta-Analyses (PRISMA) [14], and Assessing the Methodological Quality of Systematic Reviews (AMSTAR 2) [15] guidelines. Our study was prospectively registered on the PROSPERO database (registration number: CRD42024595324), and no prior ethical approval was required to conduct this review.

### Search strategy and study selection

We performed an online search of four electronic databases: Medline, PubMed, Embase, and Cochrane. These data sources were interrogated from inception to 27th September 2024 for all comparative studies of adult patients (> 18 years) with colostomy or previous HP requiring a reversal, either through conventional, multi-port laparoscopy (CL) or single-port laparoscopic reversal (SILS). Our intervention group was SILS and the comparator was CL.

Keywords used in our search included the following: “single-port” OR “single incision” AND “multiport” OR “conventional” OR AND “Colostomy reversal” OR “colostomy closure” OR “Hartmann’s reversal.” Our primary outcomes were operative duration and post-operative mortality rate. Our secondary outcomes were post-operative complications (overall and Clavien-Dindo (CD) grade 3 + complications), length of hospital stay (LOS), re-operation rate, and intra-operative visceral injury. Two authors independently performed the search, title screening, and full-text screening before inclusion. A third author was consulted to resolve any discrepancies arising during this process.

### Inclusion and exclusion criteria

Only comparative observational or experimental studies of SILS and CL were included. We specifically excluded studies comparing open surgery reversal, published protocols for clinical trials, single-arm studies, case reports and case series, conference abstracts, online posters, reviews and editorials. No restrictions were applied on language or geographical region.

### Data extraction

A Microsoft Excel (Microsoft, v13.5) spreadsheet was created and pilot-tested for data extraction [16]. Data extracted was categorised for both SILS and CL based on study-related data (name of first author, country of origin, title of study, population in each arm, intervention, study inclusion/exclusion criteria, outcomes, and funding), baseline demographics (average age, male gender, smoking, average body mass index (BMI), and American Society of Anaesthesiologists (ASA) 3 + score) and clinical outcomes (mortality, conversion to open surgery, operative duration, visceral injury, failure of procedure, time to first stool, LOS, overall postoperative complications, Clavien Dindo 3 + complications, re-operation, anastomotic leak, bowel obstruction, wound dehiscence, peritonitis, estimated blood loss, and formation of diverting ileostomy).

These are summarized in Tables 1, 2 and 3, respectively.

**Table 1:** Study-related data for individual studies

Author	Year	Country	Journal	Type of study	Population	Interventions	Exclusion Criteria	Outcome	Funding
Thambi et al. [20]	2019	United Kingdom	Colorectal disease	Retrospective, cohort	Patients who had undergone elective Hartmann’s reversal between February 2007 and February 2017 using single-port technique	SP-HR (56/72), CL-HR (16/72)	N/A	Basic demographics, intraoperative outcomes and morbidity, postoperative outcomes and morbidity	None
D'Alessandro et al. [21]	2020	France	Techniques in coloproctology	Case-controlled study	Between December 2013 and November 2017, patients underwent single-port Hartmann’s reversal case-matched to those undergoing multiport laparoscopic variant	SP-HR (44/88), CL-HR (44/88)	N/A	Basic demographics, intraoperative outcomes and morbidity, postoperative outcomes and morbidity	N/A

*SP* single-port, *HR* Hartmann’s reversal, *CL* conventional laparoscopy

**Table 2:** Patient demographics in included studies

Author	Number of patients	SP-HR	CL-HR	Age (yrs) (mean/median, SD or range)	Male gender (*n*, %)	Smoking	Average BMI	ASA III +
Thambi et al. [20]	72	56	16	62 (32–87) vs. 54 (44–81)	34 (61%) vs (69%)	N/A	29 (21–42) vs. 26 (18–35)	20 (36%) vs. 3 (19%)
D’Alessandro et al. [21]	88	44	44	62 ± 12.9 vs. 59 ± 13.8	30 (68%) vs. 30 (68%)	N/A	24.2 ± 5.5 vs. 23.2 ± 4.9	4 (11%) vs. 5 (11.4%)

*SP* single port, *HR* Hartmann’s reversal, *SD* standard deviation, *N* number, *BMI* body mass index, *ASA* American Society of Anaesthesiologists

**Table 3:** Summary of outcomes reported by individual studies

Author	Patients	Mortality	Conversion to open	OP duration	Visceral injury	Failure of procedure	Time to first stool	LOS	Overall postop complications	Clavien Dindo 3 +	Re-operation	Leak	BO	Blood loss	Diverting ileostomy
Thambi et al. [20]	SP-HR: 56, CL-HR: 16	1 (1.7%) vs. 0 (0%)	5 (8.9%) vs. 6 (37.5%)	**182 ± 99.6 vs 213 ± 60.9 (*P* < 0.001)**	6 (10.7%) vs 0 (0%)	1 (1.8%) vs. 0 (0%)	**5 ± 3.4 vs. 5 ± 2.6 (*P* < 0.001)**	**13.5 ± 12.2 vs. 12.3 ± 8.9 (*P* < 0.001)**	17 (30.4%) vs. 7 (43.8%)	5 (8.9%) vs. 2 (12.5%)	2 (3.6%) vs. 0 (0%)	N/A	1 (1.7%) vs. 0 (0%)	N/A	0 (0%) vs. 0 (0%)
D'Alessandro et al. [21]	SP-HR: 44, CL-HR: 44	1 (2.2%) vs. 0 (0%)	0 (0%) vs. 0 (0%)	105 ± 52.3 vs. 155 ± 63.7	2 (4.5%) vs. 2 (4.5%)	0 (0%) vs. 0 (0%)	N/A	4.8 vs. .6.8	7 (15.9%) vs. 10 (22.7%)	1 (2.2%) vs. 2 (4.5%)	0 (0%) vs. 1 (2.2%)	1 (2.2%) vs. 2 (4.5%)	N/A	115 ± 69.3 vs. 185 ± 115.5	1 (2.2%) vs. 1 (2.2%)

*SP* single port, *CL* conventional laparoscopy, *OP* operation, *LOS* length of stay, *BO* bowel obstruction

### Quality assessment

This review included observational studies only and consequently the star-based scoring system—the Newcastle–Ottawa Scale (NOS) was used [17]. This system assesses the selection, comparability, and ascertainment of exposure in the studied cohorts. A score of 6 or less deems the study to be at high risk of bias (Tables 4 and 5).

**Table 4:** Newcastle–Ottawa quality assessment scale for cohort studies—Thambi et al

Study	Selection				Comparability	Outcomes			Total
	Representativeness of the exposed cohort	Selection of the non-exposed cohort	Ascertainment of exposure	Outcome not present at the start of the study		Assessment of the outcome	Was follow-up long enough	Adequacy of follow-ups	
Thambi et al. [20]	*	*	*	*		*		*	6

**Table 5:** Newcastle-Ottowa quality assessment scale for case–control studies—D’Alessandro et al

Study	Selection				Comparability	Outcomes			Total
	Adequacy of case definition	Representativeness of cases	Selection of cases	Definition of controls		Ascertainment of exposure	Same method of ascertainment for cases and controls	Non-response rate	
D’Alessandro et al. [21]		*		*	**	*	*		6

### Statistical analysis

Statistical and meta-analysis were performed using Cochrane Review Manager [18] (RevMan) (computer program), version 7.2 (Cochrane Collaboration, 2024) based on a random-effects model using the Mantel–Haenszel method. Cochrane *Q* test was used to assess study heterogeneity and quantified using the (*I*^2^) statistic: no heterogeneity at 0%, low heterogeneity at 25%, moderate heterogeneity at 50%, and high/substantial heterogeneity at > 75%.

Forest plots were generated to visually represent outcomes reported by two or more studies with the same variables and units. Dichotomous variables were reported as Odds Ratio (OR) with 95% confidence interval (CI). Continuous variables were reported as mean differences (MD) with 95% CI. The mean and standard deviation (SD) were extrapolated (where necessary) from the median and range of the original data using a validated formula by Hozo et al. [19] Categorical data was reported in the form of percentages or frequency. A *P*-value of < 0.05 was used as a cut-off for statistical significance in this review.

## Results

### Demographics

Following the screening of articles, only two studies met our inclusion criteria [20, 21]; one retrospective cohort study [20] and one case–control study [21]. The article by van Loon et al. [12] was omitted from our review despite the authors reporting outcomes for both single and multiport surgery; their original cohort was intended to analyse for single-port procedures only, and the subsequent multi-port analysis was based only on patients requiring intraoperative conversion from single-port laparoscopy—rather than a separate comparative cohort.

**Fig. 1:**
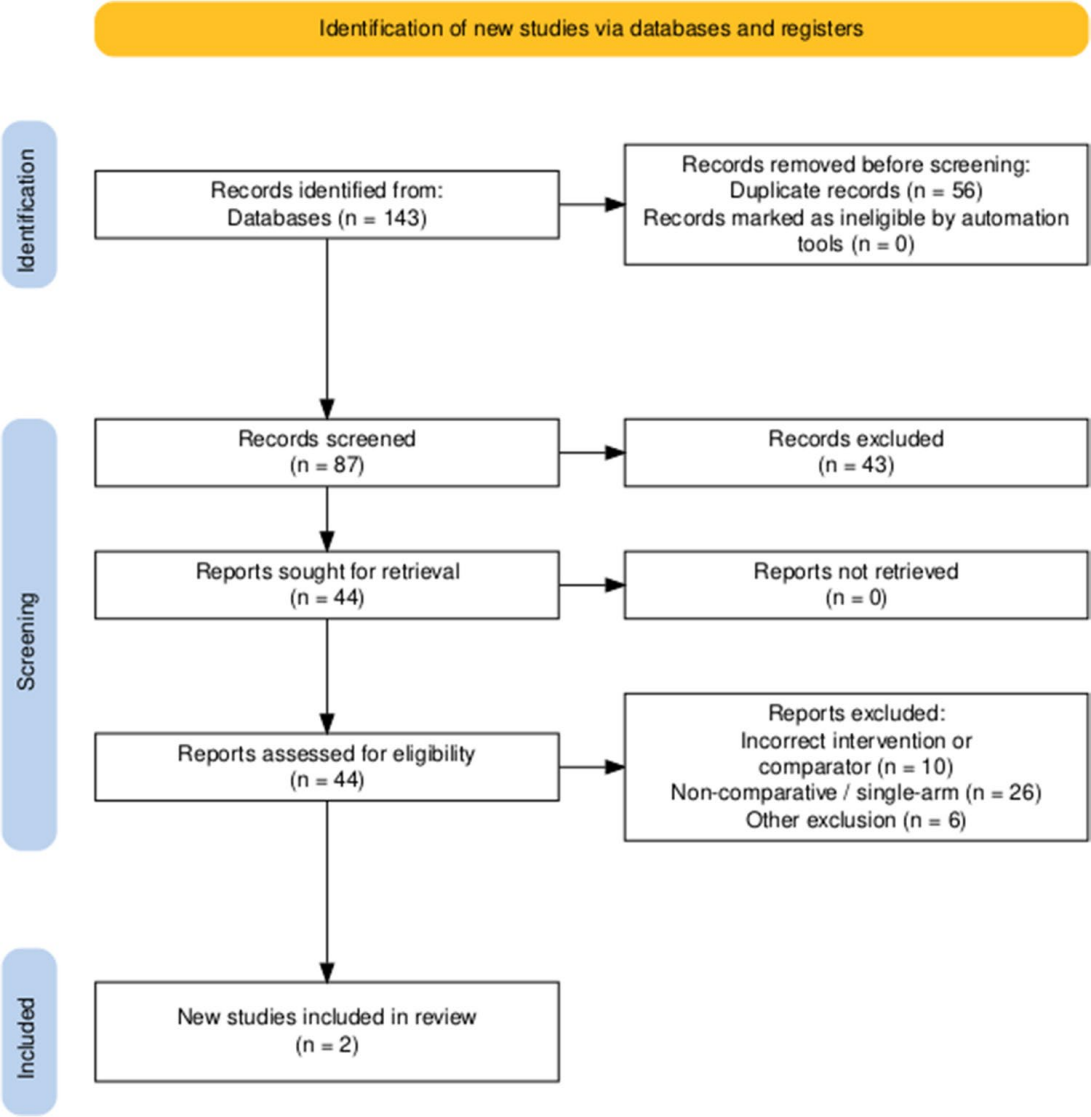
PRISMA flow chart

**Fig. 2:**
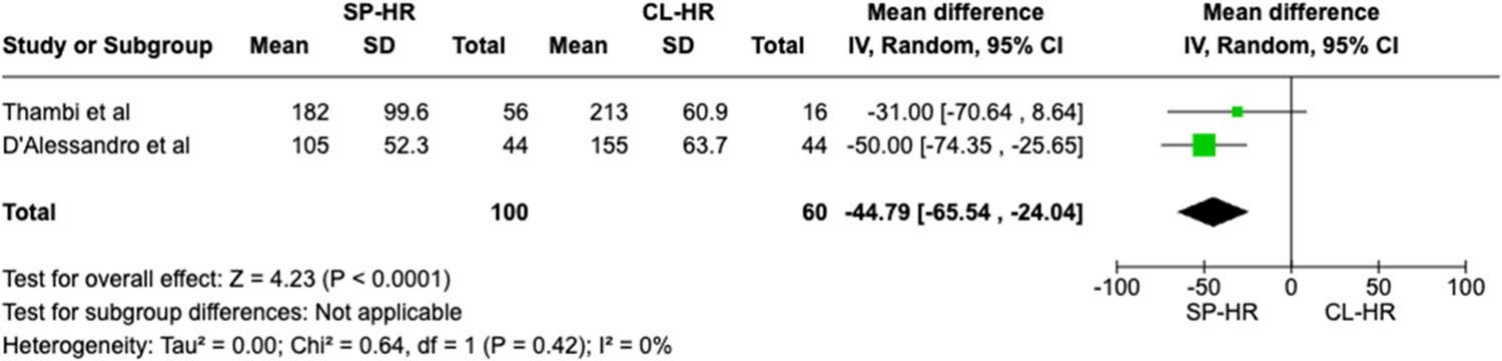
Forest plot of operative duration for single-port (SP) Hartmann reversal (HR) vs. conventional laparoscopy (CL)

**Fig. 3:**
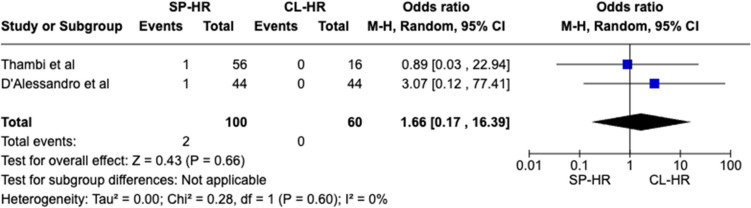
Forest plot of mortality rate for single-port (SP) Hartmann reversal (HR) vs. conventional laparoscopy (CL)

**Fig. 4:**
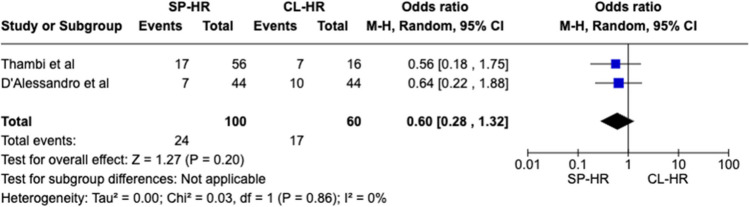
Forest plot of overall post-operative complications for single-Port (SP) Hartmann reversal (HR) vs. conventional laparoscopy (CL)

**Fig. 5:**
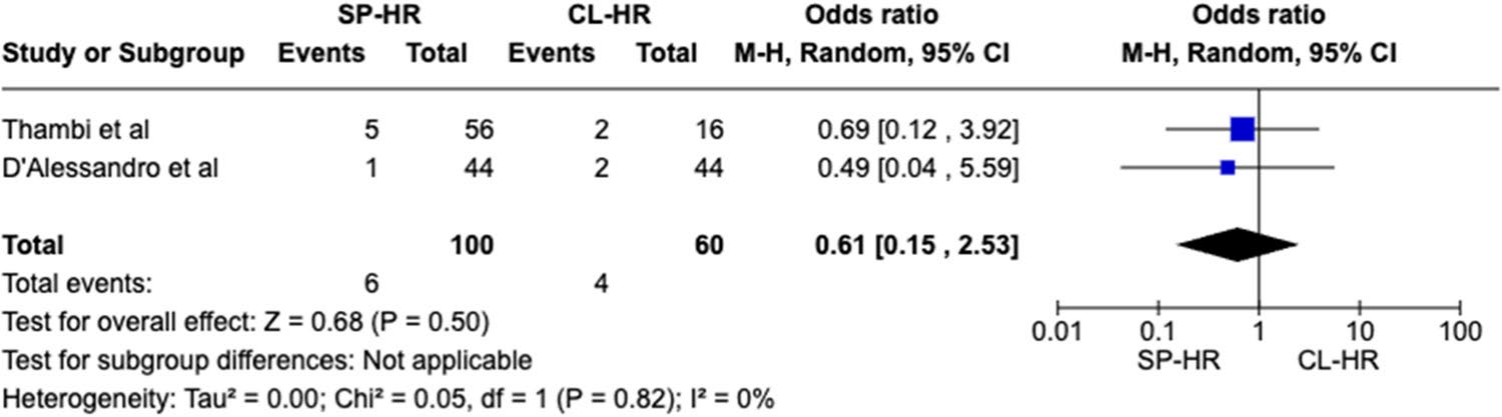
Forest plot of Clavien-Dindo 3 + complications for single-port (SP) Hartmann reversal (HR) vs. conventional laparoscopy (CL)

**Fig. 6:**
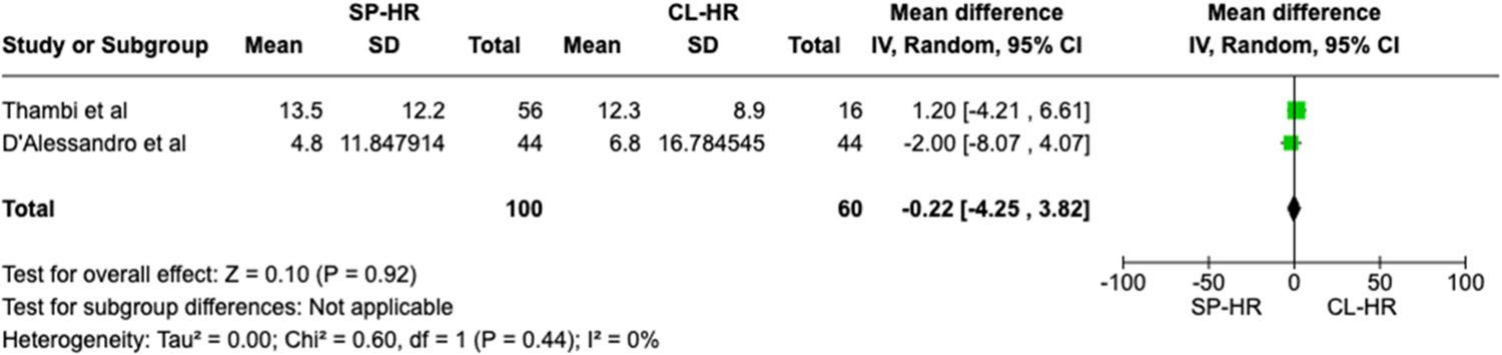
Forest plot of length of hospital stay for single-port (SP) Hartmann reversal (HR) vs. conventional laparoscopy (CL)

**Fig. 7:**
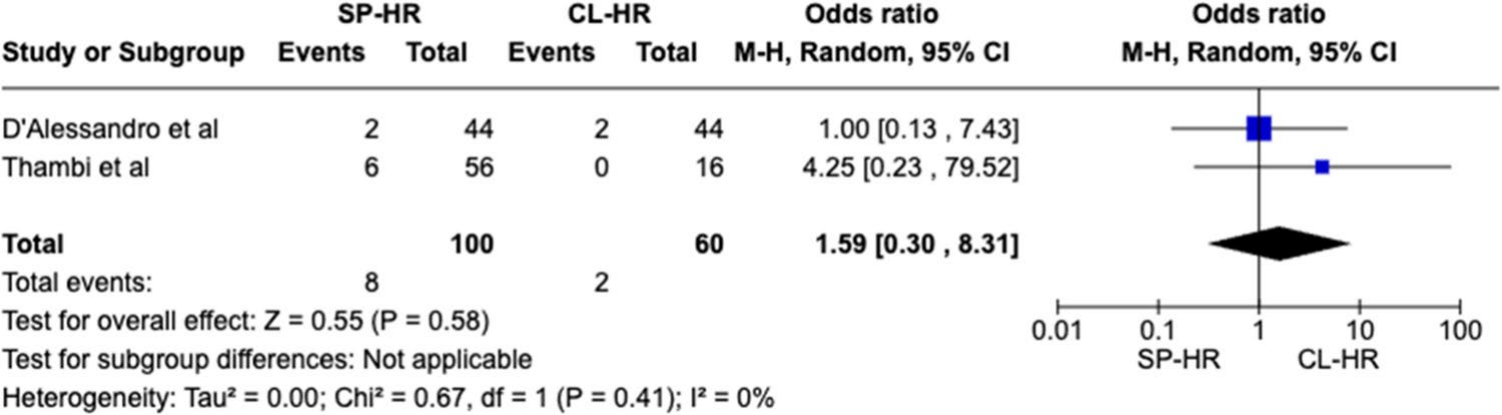
Forest plot of visceral injury for single-port (SP) Hartmann reversal (HR) vs. conventional laparoscopy (CL)

**Fig. 8:**
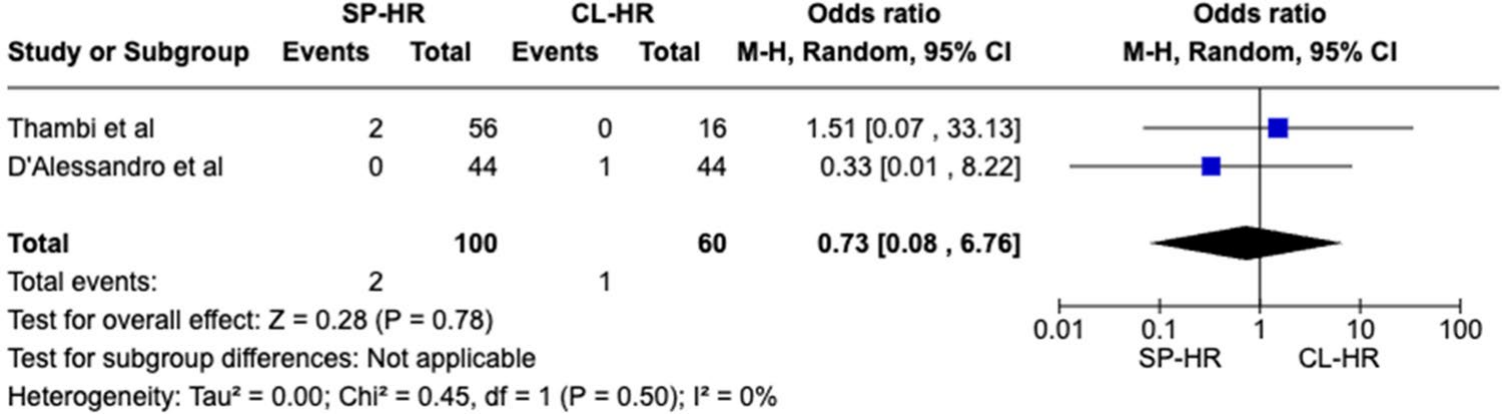
Forest plot of re-operation rate for single-port (SP) Hartmann reversal (HR) vs. conventional laparoscopy (CL)

A total of 160 patients were included in the meta-analysis, of which 100 patients formed the SILS group and 60 in the CL group. The average age was 62 years (range 32–87) in the SILS group, and 56.5 years (range 44–81) in the CL group. The summary of our search is provided in Fig. 1 and summary of patient demographics across the included studies is provided in Table 2. Primary and secondary outcomes are shown in Figs. 2, 3, 4, 5, 6, 7, and 8.

### Primary outcomes

#### Operative duration

There was a statistically significant difference observed between the two cohorts, favoring the SILS group (MD − 44.79 CI − 65.54– − 24.04, *P* < 0.0001). Cochran *Q* test detected no between-study heterogeneity (*I*^2^ = 0%, *P* = 0.42) (Fig. 2).

#### Mortality rate

No significant difference was observed for this outcome between the two cohorts (OR 1.66 CI 0.17–16.39, *P* = 0.66). There was no between-study heterogeneity reported (*I*^2^ = 0%, *P* = 0.60) (Fig. 3).

### Secondary outcomes

#### Overall postoperative complications

No significant difference was observed between the two cohorts for overall post-operative complications (OR 0.60 CI 0.28–1.32, *P* = 0.20). Cochran *Q* test revealed no between-study heterogeneity (*I*^2^ = 0%, *P* = 0.86) (Fig. 4).

#### Clavien Dindo 3 + complications

No significant difference was observed for this outcome measure between the two groups (OR 0.61 CI 0.15–2.53, *P* = 0.50). Cochran *Q* test revealed no between-study heterogeneity (*I*^2^ = 0%, *P* = 0.82) (Fig. 5).

#### Length of hospital stay

No significant difference was seen in the length of hospital stay (LOS) between the SILS and CL group (MD − 0.22 CI − 4.25–3.82, *P* = 0.92). No between-study heterogeneity was detected (*I*^2^ = 0%, *P* = 0.44) (Fig. 6).

#### Visceral injury

For intra-operative visceral injury, no significant difference was observed between our two comparison groups (OR 1.59 CI 0.30–8.31, *P* = 0.58). There was no heterogeneity detected between the included studies (*I*^2^ = 0%, *P* = 0.41) (Fig. 7).

#### Re-operation rate

For this analysed outcome, no significant difference was reported between the two techniques (SILS vs. CL) (OR 0.73 CI 0.08–6.76, *P* = 0.78). Cochran *Q* test found no between-study heterogeneity (*I*^2^ = 0%, *P* = 0.50) (Fig. 8).

## Discussion

To our knowledge, this is the first review exploring and pooling the results of available comparative studies between two laparoscopic techniques: SILS and conventional multi-port laparoscopy for HR. The single-port approach seems to be associated with a shorter operative duration compared with the traditional multi-port procedure, but no difference was seen in any of the other analysed intra- or post-operative outcomes between the two minimally invasive techniques.

The feasibility and safety of laparoscopic techniques in HR is evidently superior to open surgery, but the advantages of multi-port compared with single-port approaches remains unclear. Stoma formation, complications, and reversal are associated with significantly high morbidity, mortality, and psychological burden [22]. Thus, an efficacious approach to reversal should result in improved psychological well-being and early restoration of bowel function with minimal short-, mid-, and long-term complications.

The evolution of minimally invasive surgery has raised the question of appropriate stoma formation and reversal techniques, particularly with the emergence of robotic surgery [23]. Since Smith and Bettinger’s [24] first case-report on single-port laparoscopy, the literature on this particular surgical technique remains sparse, with variable reporting of intra- and post-operative outcomes. Our review found no difference across the analysed outcomes except operative duration.

This finding is possibly explained by the direct approach in placing the single port, using the pre-existing stoma site and avoiding additional operative time in creating new wound incisions and closure. Furthermore, the colostomy site itself is an anatomical site through which the port can be inserted, avoiding additional ports and their complications [25]. The case report by Smith and Bettinger reported a mean operative duration of 104 min with no long-term complications and a LOS of 5 days [24].

Van Loon et al. [12] performed a retrospective, multi-center audit of the single-port technique in 156 patients, reporting a procedure success of up to 98%, a median LOS of 4 days and a complication rate of less than 30%, with no reported mortality. Their average operative time was 132 min. Furthermore, they also reported similar findings in another retrospective single-centre analysis; less than 10% of patients had Clavien-Dindo grade 3 or higher complications and a median LOS of three days [26]. Other single-arm studies have also reported similar LOS, shorter duration of surgery, and no significant post-operative morbidity or mortality [8, 27].

Patients undergoing HR often have extensive intra-abdominal adhesions. They may also have concomitant defects in the abdominal wall that affects port placement, thus increasing the risk of visceral injury and conversion to multi-port or open colostomy reversal. The rates of visceral injury and conversion remain non-concordant with the literature. In a retrospective analysis of 12 patients undergoing SILS, van Loon et al. [28] reported that 50% of patients were converted to multi-port surgery with no post-operative mortality. In a further comparative study of 72 patients, Thambi et al. [20] reported 8.9% and 37.5% risk of conversion to open from a single-port and multi-port approaches, respectively.

D’Alessandro et al. reported a 4.5% incidence of visceral injury amongst both cohorts, with no cases being converted to open or multiport. Van Loon et al. reported 17 of 85 patients (20%) undergoing a SILS reversal were converted to multiport. Our review found no difference between the two groups in the incidence of visceral injuries intraoperatively, but we could not estimate a pooled effect size for conversion to open due to limited data availability. Therefore, we call for robust studies to establish a consensus on using SILS for HR and wider colorectal resections.

Restoration of gastrointestinal function is a challenge that impacts patients’ physical and psychological wellbeing [29]. Thambi et al. [20] reported an average of five days for return of bowel function in both single-port and multi-port techniques (*P* < 0.001) with no requirement for a diverting ileostomy in any of the cases. However, they reported bowel obstruction in one case (1.8%) in the SILS group requiring re-operation.

D’Alessandro et al. [21] reported a single case of diverting ileostomy in each cohort (2.2%) of 44 patients but did not report on the return of bowel function. In a single-arm cohort study of 22 consecutive patients undergoing SILS HR, Choi et al. [25] reported a median of three days for time to first flatus and 4.5 days to first solid diet with one case (4.5%) of postoperative ileus. In the present study, we were unable to synthesise data on return of bowel function thereby limiting our conclusions.

Furthermore, the impact of single-port surgery on improving patients’ psychological wellbeing remains unexplored and arguably overlooked. The overall impact of stoma reversal is positive, irrespective of surgical technique. Huang et al. [30] reported improved quality of life (QoL) following HR after 13 years in a 65-year-old male with an intestinal fistula complicating the stoma.

Theoretically, SILS surgery (minimising ‘access trauma’) performed through the existing colostomy site should help cosmesis, limit scarring and/or incidence of future port site/incisional hernias compared with a conventional multiport approach [8]. Additional benefits may also include reduced incidence of post-operative pain and analgesic requirements allowing earlier mobilisation. Given the paucity of data on QoL parameters, we argue for more qualitative studies to evaluate the impact of single-port techniques on the return of physical and psychological function.

## Limitations

The principal limitation of this review is the overall lack of comparative observational or experimental studies; only two studies identified were eligible for a meta-analysis with small population size. Although not ideal, a meta-analysis was performed as per the Cochrane Handbook of Systematic Reviews [13]. Secondly, the significant heterogeneity in baseline patient demographics, methodology, lack of adequate follow-up, and thus lack of long-term data between the two included studies introduces numerous biases including selection bias. However, the overall quality assessment was good across both studies. Thirdly, the limited literature has precluded us from reviewing and evaluating several minimally invasive techniques, such as SILS versus robotic HR. Finally, some outcomes were not amenable to having an estimated OR or mean difference, which resulted in outcomes being omitted from the meta-analytical process.

Despite these methodological weaknesses, this is the first systematic review and meta-analysis attempting to compare two laparoscopic interventions (single-port vs. multi-port) for colostomy reversal. The findings of this review highlight a significant gap in the literature that needs to be addressed with well-designed, adequately powered studies to evaluate outcomes of various minimally invasive techniques with a view to providing optimal patient care.

## Conclusion

Accounting for study limitations, the SILS procedure seems to be quicker with non-inferior outcomes compared with the conventional multi-port laparoscopic approach for HR. SILS may offer better patient satisfaction, improved cosmesis, and potentially reduce long-term complications. However, methodologically robust observational and experimental studies are warranted to compare short and long-term outcomes of patients undergoing single-port versus multi-port laparoscopy.

## Data Availability

No datasets were generated or analysed during the current study.
